# The Human Visual Claustrum Responses to Physical Stimulus Properties and Subjective Content During Movie Viewing

**DOI:** 10.1002/hbm.70583

**Published:** 2026-06-25

**Authors:** Adam Coates, Paul Sedlmayr, Anna Wastian, Hannes Mayrhofer, David Linhardt, Christian Windischberger, Andreas Bartels, Anja Ischebeck, Natalia Zaretskaya

**Affiliations:** ^1^ Department of Psychology University of Graz Graz Austria; ^2^ BioTechMed‐Graz Graz Austria; ^3^ High Field MR Center, Center for Medical Physics and Biomedical Engineering Medical University of Vienna Vienna Austria; ^4^ Werner Reichardt Centre for Integrative Neuroscience University of Tübingen Tübingen Germany

## Abstract

The human claustrum is a small bilateral grey matter structure that is highly interconnected with cortical and subcortical regions. It has been implicated in different functions including sleep, multisensory integration, consciousness and attention, yet its exact function remains unclear. The primate claustrum is known to have distinct sensory regions, with the visual zone recently demonstrated in humans using high‐resolution fMRI. In this study, we investigated stimulus properties that drive human visual claustrum activity. First, we tested the association of its response with various low‐ and mid‐level physical stimulus features, including temporal and spatial contrast, color and motion. Second, we tested the association with subjective ratings of arousal, valence and interest. To compare the claustrum's responses with visual cortical regions, we performed the same analysis with the hV4 and hMT/V5+ complex. We found that the claustrum's visual response was associated with motion, as well as with arousal, interest and valence. The pattern of claustrum responses was similar to hMT/V5+. Given the well‐established link between arousal and attentional allocation, as well as between saliency and motion, our results suggest that the visual claustrum may contribute to saliency detection and attention modulation during the sensory input.

## Graphical abstract

To determine what drives the responses of the visual claustrum zone, we extracted physical features and collected participants' subjective ratings of naturalistic videos. Claustrum activity was associated with motion, but also with subjective ratings of arousal, valence, and interest, implying the human claustrum's role beyond sensory processing.

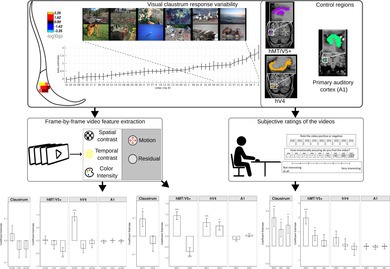

## 1 Introduction

The claustrum is a thin sheet‐like structure situated between the putamen and insula. The claustrum is unique because it is connected to most regions of the cortex, including sensory cortices and the frontal cortex (Fernandez‐Miranda et al. 2012; Pathak and Fernandez‐Miranda 2014; Lei et al. 2025; Sloniewski et al. 1986; Torgerson et al. 2014; Wendt et al. 2024). The claustrum's widespread reciprocal connections with the cortex make it a suitable candidate for various functions including sleep (Atlan et al. 2024; Narikiyo et al. 2020), attention modulation (Atlan et al. 2018; Goll et al. 2015; White et al. 2018), cognitive control (Mathur 2014), consciousness (Crick and Koch 2005), and sensory integration (Gattass et al. 2014; Remedios et al. 2010). Despite many hypotheses, the exact function of the claustrum remains unclear.

A key feature of the claustrum's functional organization in mammals is the presence of the distinct sensory zones that have reciprocal projections to corresponding cortical areas (Gattass et al. 2014; Goll et al. 2015; Lei et al. 2025; Olson and Graybiel 1980; Remedios et al. 2010; Sloniewski et al. 1986). Visual and auditory claustrum zones contain neurons with unimodal sensory responses of the corresponding modality (Remedios et al. 2010). In a recent human 7 T fMRI study (Coates et al. 2024), we identified visual claustrum activity in response to naturalistic video stimuli at a location consistent with the visual zone observed in primates (Remedios et al. 2010). However, since these videos varied widely in content, it remained unclear which stimulus features drive the claustrum responses.

Animal studies suggest that both physical stimulus characteristics and subjective aspects of the stimuli may modulate claustrum responses. For example, neurons in the visual claustrum of cats respond preferentially to elongated, moving stimuli (Sherk and LeVay 1981). Research on auditory claustrum neurons in the macaque revealed transient responses to sound onsets and changes, suggesting a role in detecting salient events (Remedios et al. 2014). Recent rodent research further supports a role of the claustrum in attention and salience processing. It is reciprocally connected with the anterior cingulate cortex (ACC), a region central to selective attention. According to one hypothesis, the claustrum filters noisy input and enhances task‐relevant sensory signals, transmitting them to frontal regions to support attentional focus (Goll et al. 2015). Disrupting this circuit impairs distractor resistance and task performance (Atlan et al. 2018), and serotonergic modulation may further influence these attentional functions (Alves et al. 2022; Goll et al. 2015).

These findings suggest that the claustrum may contribute to the salience network (SN), integrating sensory and emotional information to guide behavior. Its strong bidirectional connections with prefrontal areas, particularly the ACC, support this view (Qadir et al. 2018; Smith et al. 2019; White et al. 2018). Claustrum responses to attentional salience also appear to be modulated by the emotional properties of stimuli. Its dense interconnections with the limbic system, especially the basolateral amygdala (BLA), suggest a mechanism for integrating affective information to enhance attention toward emotionally salient stimuli (Atlan et al. 2018; Mathur 2014; Smith et al. 2019). For example, aversive sounds increase activity in both the temporal claustrum and lateral amygdala (Zald and Pardo 2002), and stimulation of claustral neurons receiving BLA input elevates anxiety‐related behavior in mice (Niu et al. 2022). These findings underscore the claustrum's potential role in integrating emotional salience with attentional control. Together, this evidence highlights the claustrum's dual role in sensory and attentional processing. What remains unclear is whether sensory and emotionally‐ or salience‐driven responses in the claustrum remain segregated, or whether signatures of salience can be detected in the sensory zones.

In the current study we addressed this question by investigating the association between fMRI activity of the visual claustrum zone and physical as well as subjective content of natural video stimuli. We extracted low‐ and mid‐level visual features (e.g., luminance, contrast, motion) and collected independent behavioral ratings of valence, interest, and arousal. We then used data from Coates et al. (2024) to test how each of these variables relates to claustrum activation across stimuli.

## 2 Methods

### 2.1 Claustrum Responses to Individual Videos

To determine whether the visual claustrum showed a preference for specific physical stimulus features or subjective ratings, we took advantage of an ultra‐high‐field high‐resolution fMRI dataset previously published in Coates et al. (2024), and analyzed claustrum responses to the individual naturalistic videos (see https://doi.org/10.17605/OSF.IO/WPA4V for examples). The details of the experiment are presented briefly in this section.

#### 2.1.1 Participants

12 (mean age = 24.50 years, SD = 3.66 years, 9 females and 3 males, 11 right‐handed) subjects scanned across 2 separate sessions on two separate days were included in the current analysis.

#### 2.1.2 Data Acquisition and Preprocessing

Data were acquired using an ultra‐high field 7 Tesla Siemens MAGNETOM scanner (Siemens Healthineers, Erlangen, Germany) with a 32‐channel head coil (Nova Medical, Wilmington, MA, USA). Functional images were acquired using a partial slice prescription coverage of either the left or the right claustrum. We acquired 37 sagittal slices at 1.34 mm × 1.34 mm resolution with a slice thickness = 0.8 mm (TR = 2000 ms; TE = 23 ms; FA = 62°, GRAPPA acceleration factor = 2), resulting in a slab of 29.6 mm width. For more details about the anatomical and functional scanning parameters see Coates et al. (2024). Participants were shown the same 15‐s naturalistic video clips as either visual only, auditory only, or audiovisual conditions and an additional baseline (no stimulus) and were instructed to press a button during a central fixation task.

For the anatomical preprocessing we first removed extracerebral noise from the MP2RAGE scan and then combined the MP2RAGE scans from both sessions using *mri_robust_template*. The scans were then segmented using the CAT12 pipeline (Dahnke and Gaser 2017) to generate a high‐resolution brain mask. We then performed autorecon1 from FreeSurfer's *recon‐all* (Dale et al. 1999; Greve and Fischl 2009) command and substituted the CAT12 derived mask for the automatically generated FreeSurfer mask. We then continued with *recon‐all* until completion of autorecon2 and 3. Functional data were preprocessed using FreeSurfer's FSFAST stream. We motion corrected the scans and then performed distortion correction using FSL's *topup* and *applytopup* (Andersson et al. 2003) commands. We then co‐registered the functional and anatomical scans using boundary‐based registration with 6 degrees‐of‐freedom using the command *bbregister* (Greve and Fischl 2009). Functional data were then resampled to the anatomical scan. For the following analysis functional data remained in the subject space. We visually inspected the alignment between functional EPI images and the corresponding anatomical T1‐weighted images for each participant after coregistration (see Figure S1). We also checked the tSNR values in the claustrum, the surrounding regions (putamen and insula), the visual regions hMT/V5+ and hV4, and the control primary auditory cortex region. This showed comparable tSNR values across regions, suggesting adequate signal quality in the claustrum (Figure S2).

#### 2.1.3 First‐Level General Linear Model Analyses

We first wanted to determine the response of the previously identified visual claustrum zone to each of the video stimuli. To do this, we used the native space functional data and modeled each of the 48 videos from the visual‐only condition as a separate regressor of interest in a general linear model (GLM) analysis separately for each session. Each movie regressor hence consisted of a single HRF‐convolved box‐car, specific to each movie. Run‐specific offsets, scanner drifts (modeled with a quadratic polynomial term), and the first 4 timepoints (to ensure that the scanner reached magnetic equilibrium) were modeled as nuisance regressors. The resulting beta coefficients for each video and session were extracted from the regions of interest and used as the dependent variable for the subsequent linear mixed model analyses. In this per‐video GLM, no explicit baseline condition was modeled. Therefore, each video beta estimate reflects activation relative to the implicit baseline (corresponding to no stimulation periods).

To address the possibility that the hemodynamic response function (HRF) in the claustrum may differ from the canonical shape, we performed an additional finite impulse response (FIR) analysis. The FIR model estimates the hemodynamic response at each time point without assuming any particular shape, providing a model‐free comparison against the predicted responses derived by the convolution with canonical HRF used in the conventional GLM analysis (Coates et al. 2024). We used FreeSurfer's FSFAST pipeline (time window: 0–14 s, temporal estimation resolution: 2 s, matching the TR), yielding 7 time bins (bin centers at 1, 3, 5, 7, 9 and 11 s) with corresponding 7 FIR regressors per condition. Time window below 15 s (video duration) ensured that each condition's hemodynamic response was estimated independently, preventing overlap with adjacent conditions in the continuous stimulus presentation design. The resulting FIR beta estimates were extracted from the claustrum ROI for the visual‐only condition and compared against the predicted response generated by convolving the canonical SPM double‐gamma HRF with a 15 s stimulus boxcar function, sampled at the 7 time points relative to the stimulus onset. Shape agreement was quantified using Pearson correlation over time points. To assess the consistency of correlation in our sample, per‐session correlation coefficients were Fisher z‐transformed and submitted to a one‐sample *t*‐test against zero, testing whether claustrum HRF shapes were consistent with the predicted form across *N* = 24 observations (12 subjects, 2 sessions each).

#### 2.1.4 Visual Claustrum ROI Definition

To define the visually responsive region of the claustrum we utilized data from the Human Connectome Project (HCP) 7 Tesla retinotopy dataset (Benson et al. 2018). This dataset (described in detail by Benson et al. 2018) involved 7 Tesla whole brain functional data with 1.6‐mm isotropic voxel sizes and a 1000 ms TR, which was preprocessed using the HCP preprocessing pipeline (Glasser et al. 2013). 181 participants viewed dynamic, textured patterns presented in moving apertures (wedges, rings, and bars). We carried out a GLM analysis using the rotating wedge runs of the dataset, using time points when the wedge was either in the left or in the right hemifield as left and right visual field regressors convolved with a hemodynamic response function. We then calculated a contrast “left vs. right” to compare the timepoints for when the wedge subtended the left visual field versus when the wedge subtended the right visual field (Linhardt et al. 2025). This resulted in a left versus right contrast z‐statistic map for the whole brain.

As the next step, we mapped our whole claustrum mask (Coates and Zaretskaya 2024) which was in MNI152 non‐linear 2009c asymmetric space to the MNI152 non‐linear 6 asymmetric space of the HCP retinotopy dataset. To do this, we used ANTs v.2.6.0 *antsRegistrationSyN* (Avants et al. 2008, 2011; Tustison and Avants 2013). We then transformed our claustrum mask using the ANTs affine transformation matrix and diffeomorphic transformation to map our whole claustrum mask into MNI152 non‐linear 6 asymmetric space of the HCP data. Finally, we resampled our whole claustrum mask to 1.6 mm isotropic voxel size using nilearn *resample_img* (Nilearn contributors et al. 2025) to match the voxel size of the HCP retinotopy dataset. We then overlaid the whole claustrum mask onto the left versus right contrast z‐statistic map and marked voxels that were inside the whole claustrum mask that were also active in the contrast map at a threshold of z > 2.5 for the left hemisphere and z < −2.5 for the right hemisphere. Single outlier voxels not connected to the main cluster were not included in the mask.

**FIGURE 1:**
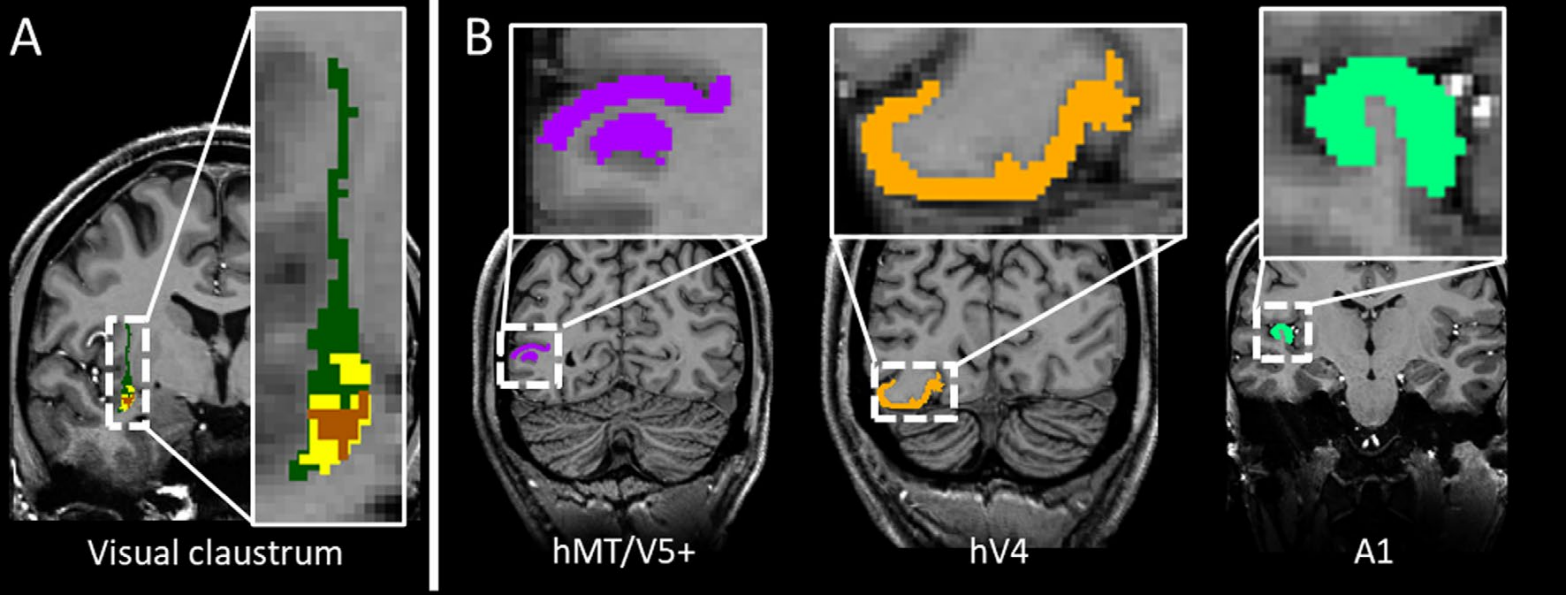
Regions of Interest (ROIs) of an example subject used to extract beta estimates. (A) The visual claustrum region shown on top of the whole claustrum label (Coates and Zaretskaya 2024) in green. The visual claustrum ROI derived from the 7 T HCP retinotopy dataset (Linhardt et al. 2025) and used in the current study is shown in yellow, and our previously identified visual claustrum zone (Coates et al. 2024) is shown in orange. (B) Control regions including hMT/V5+, hV4 and primary auditory cortex (A1).

Subsequently, the MNI space masks were transformed into the native space of each subject using the inverse deformation field generated from CAT 12 in the anatomical preprocessing step (see above). An example visual claustrum ROI of a representative participant is shown in Figure 1A. We then used these visual claustrum masks to extract beta estimates for each of the 48 videos for each participant for both session 1 and session 2, averaging over voxels in the ROI. We chose this functionally defined visual claustrum ROI because our aim was not to characterize claustrum responses across its full anatomical extent, but to test the response properties of the visual claustrum zone. At the same time, this ROI definition was more robust and better generalizable due to the large sample size of the HCP retinotopy dataset compared to our original visual claustrum mask (Coates et al. 2024), which is based on 15 subjects.

To examine differences in claustrum response across videos, we averaged the beta estimates over the two sessions and tested the response to each video against zero using a one‐sample two‐sided *t*‐test with FDR correction for 48 comparisons.

#### 2.1.5 Other Regions

To compare claustrum responses with those of other cortical regions, we included several control regions in the analysis. Since our functional data had a partial field‐of‐view covering either the left or right claustrum, visual areas hV4 and hMT/V5+ could be included as visual control regions (Figure 1B). We used the surface‐based probabilistic atlas by Wang, Mruczek, et al. (2015) to define these regions. First, we registered the atlas to the native surface space of each subject using the FreeSurfer's *mri_surf2surf* command. Then we resampled the native surface space labels into the volume space using the *mri_surf2vol* command. We then converted label files, which are a list of coordinates of voxels that are included in the label, into the NIFTI file format using the command *mri_label2vol* and finally we extracted hV4 and hMT/V5+ from the resampled atlas using the command *mri_extract_label*. This procedure yielded a single binary file for each of the respective regions of either the left or the right hemisphere.

Furthermore, to ensure that any effects we see are specific to visual responses and cannot be explained by global effects of for example, arousal on the brain, we additionally used the primary auditory cortex as a control region. The primary auditory cortex was defined using the aparc+aseg.mgz cortical parcellation volume (Dale et al. 1999; Fischl et al. 2002) generated by the FreeSurfer *recon‐all* stream. We used the *mri_extract_label* command to extract the label “superiortemporal” from the atlas which corresponds to the primary auditory cortex. Beta estimates were subsequently extracted for the control region using the same procedure as for the visual claustrum described above.

### 2.2 Physical Stimulus Feature Extraction

To characterize each video in terms of the physical stimulus feature content we used an approach described in Bartels et al. (2008) and summarized briefly below. Each feature was expressed as a single value per video, representing the maximal intensity of the respective feature within the 15‐s clip period. All features except for color were derived from a grayscale (i.e., luminance) version of each frame. Luminance values of each pixel in each frame were calculated by taking the RGB value of a pixel and multiplying it with the CIE XYZitu (D65) standard, retaining the Y‐value (luminance) only.

#### 2.2.1 Temporal Contrast

Temporal contrast quantifies the absolute change in luminance across frames. To calculate it we took the absolute difference between pairs of successive frames for each pixel and averaged over pixels for each time point. This results in an average absolute luminance difference value for each frame of the video compared to the corresponding previous frame. A single value for the whole video was calculated by taking the mean luminance difference over frames.

#### 2.2.2 Color

Color intensity was extracted using CIELAB color space that is composed of the following components: the L* component represents lightness, which is based on relative luminance, representing how bright a color appears compared to a reference white point rather than measuring absolute brightness. The a* component captures color variation along the red‐green axis, while the b* component measures color variation along the yellow‐blue axis.

To quantify color intensity per frame we used the following formula:

ColorIntensity=Luminance×Saturation

Whereby saturation is $\sqrt{a^{2}+b^{2}}$. This results in an estimate of color intensity for each frame as follows:

$$\text{ColorIntensity}=L\times \sqrt{\left(a^{2}+b^{2}\right)}$$

Similar to the other features, to get a single value for color intensity for each of the 48 videos we took the mean color intensity value over frames.

#### 2.2.3 Spatial Contrast

The root‐mean‐squared (RMS) contrast for each frame was calculated as the standard deviation of the luminance values over pixels divided by the mean luminance value. To get a single value of spatial contrast per video, we took the mean value over all frames.

#### 2.2.4 Total Motion

**FIGURE 2:**
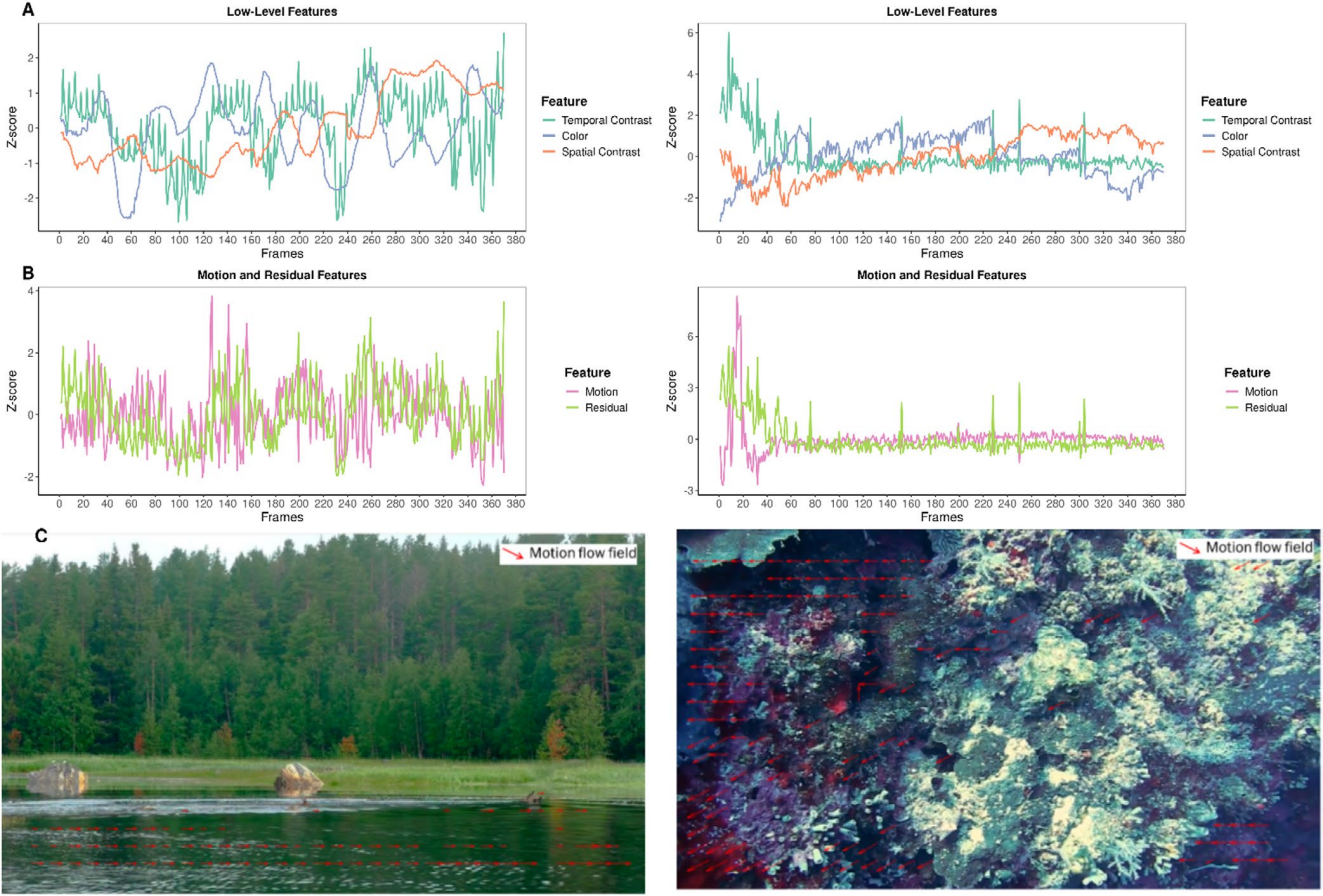
Demonstration of the frame‐by‐frame feature extraction analysis for two example clips. (A) Time course of frame‐average feature content for low‐level features. (B) Time course of frame‐average feature content for motion and residual. (C) Motion flow fields denoted by the red arrows represent motion between two adjacent frames. The length of the arrow represents the amount of motion between adjacent frames (motion velocity, corresponding to dMotionRF) for each patch and the direction of the arrow represents the direction of movement of the patch. Feature time courses in A and B were z‐scored over time for display purposes.

Motion‐related features were quantified in terms of changes in pixel luminance values between consecutive frames as described below. To quantify the amount of motion between two consecutive frames (dMotion), every frame was split into 32 × 18 receptive field (RF) patches. For each RF in a given frame (frame n), a motion vector was determined by finding its best‐matching location in the subsequent frame (frame *n* + 1). This ‘best match’ was identified by translating the RF from frame n in 12 different radial directions and 7 different distances within frame *n* + 1, selecting the translation that resulted in the minimal luminance difference between the original RF in frame n and its translated version in frame *n* + 1. This minimal difference is termed dResidualRF (the RF‐specific residual change). The total luminance difference between two subsequent frames for that RF without translation is dTotalRF. The amount of change accounted for by motion for that specific patch (dMotionRF, illustrated as red arrows in Figure 2c) was then calculated in luminance units as: dMotionRF=dTotalRF−dResidualRF. The frame‐wise dMotion and dResidual value was the average of dMotionRF and dResidualRF respectively across all RFs. To get a single value of dMotion per video we took the average difference in motion between adjacent frames.

After extracting the mean physical feature values from the videos, they were z‐standardized for the subsequent linear mixed model analysis (see below). An example of the feature extraction is shown in Figure 2.

### 2.3 Subjective Ratings

#### 2.3.1 Participants

To determine how each video is perceived subjectively by healthy adult volunteers, we recruited 68 participants who did not take part in the original fMRI experiment. Two participants were excluded due to their stimulus ratings exceeding three standard deviations from the mean, resulting in a final sample of 66 participants (M = 23.03, SD = 3.89; female = 36). Participants provided their informed consent and were compensated with course credit. The study was approved by the University of Graz ethics committee.

#### 2.3.2 Stimuli and Procedure

**FIGURE 3:**
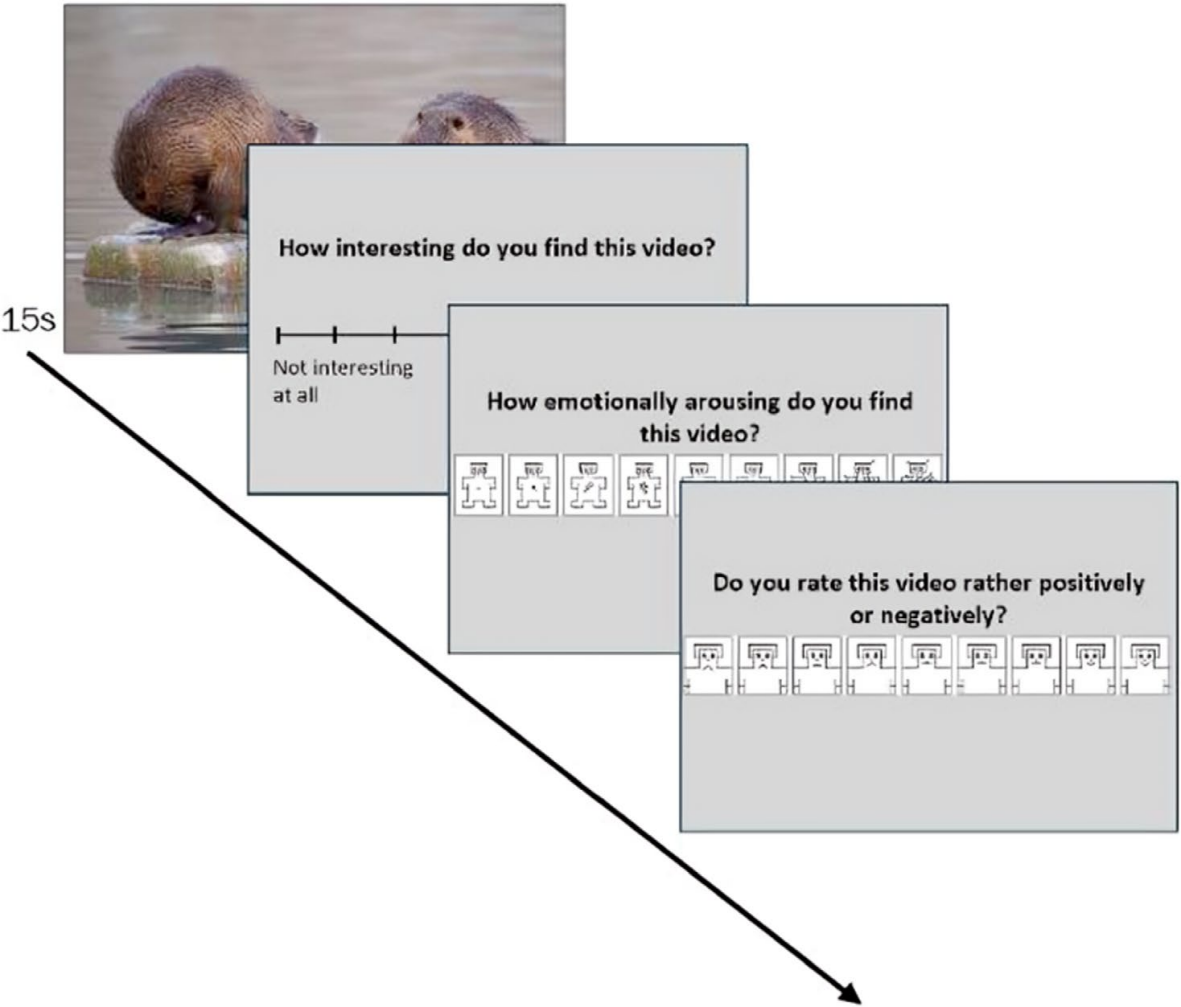
Example of a behavioral trial. Participants first saw a video for 15 s and then responded to 3 questions about their subjective experience of the videos.

Participants first took part in the experiment, which was identical to that by Coates et al. (2024), but was conducted outside of the scanner in the laboratory. During the experiment participants carried out a central fixation task whilst naturalistic audiovisual, visual and auditory 15‐s stimuli were presented in a pseudorandomized and counterbalanced order. For full details of the experiment see Coates et al. (2024). After having watched all 48 stimuli, participants took part in a free‐recall memory test (not part of the current study), followed by a rating task. In the rating task, participants were shown each of the videos one‐by‐one and were prompted to rate it in terms of interest, valence, and arousal. The current paper used the data from the rating task. The experiment was implemented using PsychoPy v2021.2.3 (Peirce et al. 2019). Stimuli were displayed on a 24″ Samsung LED display (S24C450MW, Samsung, Seoul, South Korea). During the experiment, participants viewed each of the 15‐s visual only videos from the original fMRI experiment in Coates et al. (2024) in a pseudorandom order and subsequently rated each video on three scales: valence, arousal, and interest. Each trial began with video presentation, followed by three consecutive rating questions (Figure 3). Interest was rated using a single question “How interesting do you find this video?”, with response options ranging from “Not interesting at all” (1) to “Very interesting” (8). Arousal and valence were assessed using the Self‐Assessment Manikin (SAM) scale (Bradley and Lang 1994) which contains a pictorial representation of 9 different arousal levels ranging from low to high and 9 valence values ranging from very negative to very positive. The SAM scale was chosen due to its established reliability in assessing emotional responses (Anders et al. 2008; Bradley and Lang 1994). Arousal was assessed using the question “How emotionally arousing do you find this video?”. Participants had to select the image that best represented their perceived level of arousal. Similarly, valence was rated using the item “Do you rate this video rather positively or negatively?”. The order of the three rating scales was randomized across trials. Participants provided ratings by selecting options with a mouse. Once a rating for valence, interest, and arousal was recorded, the next trial commenced, continuing until all 48 videos were rated. All videos were obtained under a Creative Commons CC0 license, permitting free use and modification. Full details on video preprocessing can be found in Coates et al. (2024).

#### 2.3.3 Preprocessing of the Scores

Each participant's raw arousal and interest ratings were first z‐standardized by subtracting the mean over videos and dividing by the standard deviation. For valence we performed an additional transformation, because the results of valence are not co‐linear with the ratings of arousal and interest (Bestelmeyer et al. 2017; Kuppens et al. 2013). A low (negative) or high (positive) rating of valence can be associated with high arousal. We therefore recoded valence values as the absolute deviation from the neutral midpoint of the scale before z‐standardization, thereby omitting the valence sign. While this means that we lose information on whether a video was rated low or high in valence, this allows us for a direct comparison with the ratings of arousal and interest. Subsequently, subjective ratings were averaged over the whole participant sample to yield one value per question and per video.

To determine to which extent subjective ratings correlate with each other, we performed pairwise Pearson's correlations between the mean ratings for arousal, interest, and valence.

### 2.4 Linear Mixed Model Analysis

To determine the relationship between claustrum responses and physical stimulus features on the one hand and subjective ratings on the other hand, we performed linear mixed model analysis (LMM), which allows to model subject‐specific random effects as well as feature or subjective rating effects in one model. Linear mixed model analysis was performed in R version 4.3.3 using the package lme4 version 1.1‐37 (Bates et al. 2015). Each participant's visual claustrum response to each of the videos in the 7 T fMRI experiment served as a dependent variable. This was taken from the first level GLM, in which each of the 48 videos were included as regressors of interest. We extracted the voxel‐averaged beta estimates for each ROI, participant, video and session. Participants of this experiment were modelled as random effects. Physical features or the subjective ratings of the stimuli were included in the model as fixed effects. The model can be expressed as $y∼x_{1}+x_{2}+\cdots +\left(1|\text{subject}\right)$, where y is the claustrum response to each of the 48 videos, $x_{1}+x_{2}+\cdots$ denote fixed effects (either physical features of the videos or subjective ratings of the videos) and $\left(1|\text{subject}\right)$ represents random effects. Two separate LMM analyses were performed for physical features (one modelling temporal contrast, spatial contrast and color, and another modelling motion and residuals). Three separate LMM analyses were performed for subjective ratings to avoid collinearity. Equivalent analysis was carried out for the regions hMT/V5+, hV4 and the primary auditory cortex. Of note, 1 participant's session was not included in the hV4 analysis as the field of view did not reach hV4.

To assess the robustness of our results, we repeated each linear mixed model analysis testing for the effects per session. In the first linear mixed model analysis, we included the claustrum's beta estimates in session 1 as a dependent variable, and in the second linear mixed model analysis, we included the claustrum's beta estimates in session 2 as a dependent variable.

## 3 Results

### 3.1 Variability in Visual Claustrum Response

**FIGURE 4:**
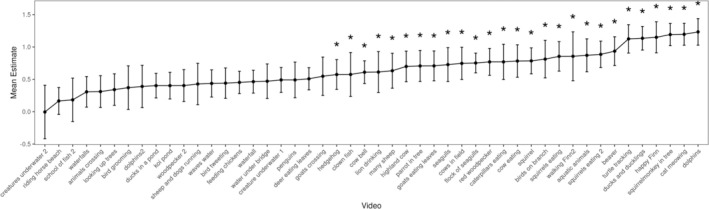
Mean and standard error of beta estimates of claustrum responses for each video in ascending order. Asterisks “*” denote a significant response compared to baseline (p_FDR_ < 0.05).

First, we tested whether there is a difference in how much each video drives the claustrum response. As Figure 4 demonstrates, we observed considerable variability in claustrum responses across different video clips. We found that 27 out of the 48 videos produced claustrum activation which was significantly greater than zero (pFDR < 0.05). This confirms that different videos drove the claustrum's responses to different extents. We therefore wanted to determine what aspects of the videos (physical features or subjective experiences) would be responsible for this variability.

To verify the appropriateness of the canonical HRF for the claustrum, we performed an additional finite impulse response (FIR) analysis. All 3 experimental conditions were modeled with separate FIR basis functions (time window: 0–14 s, bin size: 2 s), ensuring independent estimation of each condition's hemodynamic response. The model‐free FIR estimate for the visual‐only condition was compared against the predicted BOLD response that results from a convolution of the stimulus time course with the canonical SPM double‐gamma HRF. The group‐mean FIR time course (*N* = 24 sessions) showed good shape agreement with the canonical HRF (*r* = 0.82, R^2^ = 0.67). A one‐sample *t*‐test on Fisher z‐transformed per‐session correlation coefficients confirmed that the mean was significantly greater than zero (*t*(23) = 5.89, *p* < 0.001). This confirms that the canonical HRF adequately captures the hemodynamic response profile in the claustrum (see Figure S3).

### 3.2 Physical Features

#### 3.2.1 Low‐Level Feature Results

As motion‐related features are derived from low‐level features, we performed two separate linear mixed model analyses. First, we included low‐level features temporal contrast, color, and spatial contrast as fixed effects.

**FIGURE 5:**
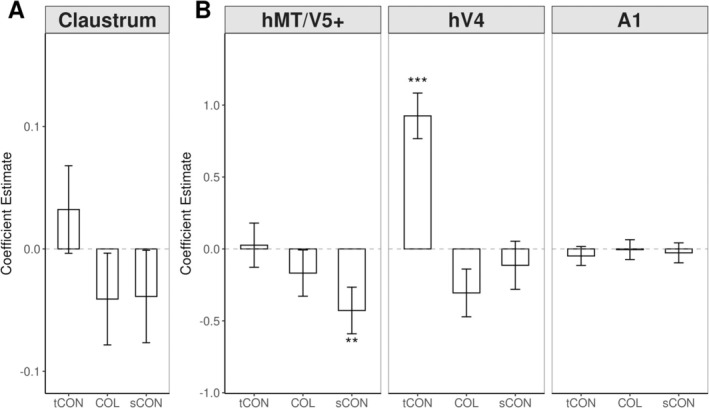
Responses to low‐level features. Linear mixed model analysis including temporal contrast (tCON), color (COL) and spatial contrast (sCON). Coefficient estimates plotted for (A) the visual claustrum and (B) cortical regions hMT/V5+, hV4 and primary auditory cortex (A1).

Claustrum responses were not significantly associated with any of the low‐level visual features (Figure 5A; temporal contrast: *t*(1146) = 0.90, *p* = 0.37; color intensity: *t*(1146) = −1.09, *p* = 0.28; spatial contrast: *t*(1146) = −1.03, *p* = 0.30). For hMT/V5+ (Figure 5B), there was a significant negative association with spatial contrast (*t*(1146) = −2.64, *p* = 0.008), but no association with temporal contrast (*t*(1146) = 0.17, *p* = 0.87) or color intensity (*t*(1146) = −1.04, p = 0.30). hV4 had a significant association with temporal contrast (*t*(1098) = 5.84, *p* < 0.001), but no significant association with color intensity (*t*(1098) = −1.84, *p* = 0.07) or spatial contrast (*t*(1098) = −0.68, *p* = 0.50). Finally, as expected, the primary auditory cortex (A1) showed no significant association with any of the low‐level visual features (all *p* > 0.05), confirming the specificity of the observed effects to the visual modality. Complete statistical information is presented in Table S1.

To assess whether these effects were consistent across sessions, we repeated the analysis separately for session 1 and session 2. This revealed a similar overall pattern of results across sessions. As in the main analysis, claustrum activity did not show any significant association with temporal contrast, color intensity, or spatial contrast in either session 1 or session 2. hMT/V5+ showed a significant negative association with spatial contrast in session 2 (*t*(570) = −2.54, *p* = 0.01) but not in session 1 (*t*(570) = −1.58, *p* = 0.12), although the direction was consistent. Additionally, hV4 showed a significant association with temporal contrast in both sessions (session 1: *t*(570) = 3.35, *p* < 0.001, session 2: *t*(522) = 5.25, *p* < 0.001; see Figure S4).

#### 3.2.2 Motion‐Related Feature Results

**FIGURE 6:**
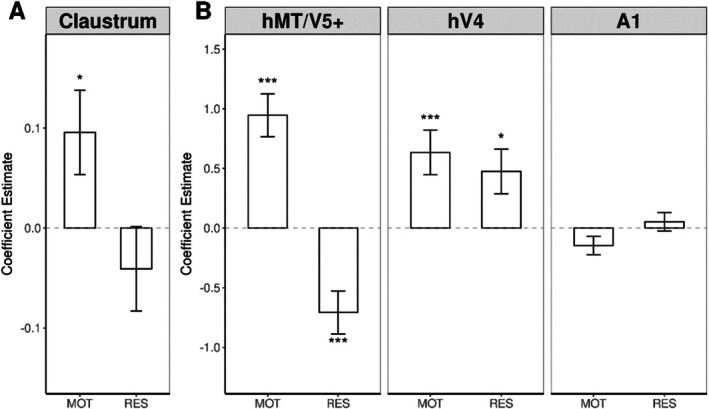
Responses to motion and residual. Linear mixed model analysis including motion (MOT) and residual (RES) (pixel differences not explained by motion). Coefficient estimates plotted for (A) the visual claustrum and (B) cortical regions hMT/V5+, hV4 and primary auditory cortex (A1).

Next, we included pixel differences that were explained by motion and pixel differences not explained by motion (residual) in a linear mixed model analysis modelling participants as a random effect. We found that the visual claustrum zone showed a significant association with motion (Figure 6A; *t*(1147) = 2.27, *p* = 0.02), but not with residual (*t*(1147) = −0.97, *p* = 0.33).

As expected and consistent with Bartels et al. (2008), area hMT/V5+ showed a significant association with motion (Figure 6B; *t*(1147) = 5.26, *p* < 0.001), as well as a significant negative association with residual pixel difference (*t*(1147) = −3.94, *p* < 0.001). Area hV4 showed a significant association with both motion (*t*(1099) = 3.39, *p* < 0.001) and pixel differences not explained by motion (*t*(1099) = 2.54, *p* = 0.01). Finally, primary auditory cortex responses were not significantly associated with motion (*t*(1147) = −1.89, *p* = 0.06) or with pixel differences not explained by motion (*t*(1147) = 0.67, *p* = 0.50). Complete statistical information is presented in Table S2.

To determine whether these effects were consistent across sessions, we repeated the analysis separately for session 1 and session 2. This revealed a similar overall pattern of results across sessions, which parallels the main analysis results. Claustrum activity was not significantly associated with either motion or residual pixel difference not explained by motion in either session, although the motion effect was similarly positive in session 1 and showed a trend in session 2 (*t*(571) = 1.85, *p* = 0.06). hMT/V5+ consistently showed a significant association with the motion feature in both sessions (session 1: *t*(571) = 4.26, *p* < 0.001, session 2: *t*(571) = 3.85, *p* < 0.001), as well as a significant negative association with residual pixel difference in both sessions (session 1: *t*(571) = −3.40, *p* < 0.001, session 2: *t*(571) = −2.64, *p* = 0.009). hV4 showed a significant association with motion in session 1 (*t*(571) = 2.84, *p* = 0.005) and with residual pixel difference in session 2 (*t*(523) = 3.13, *p* = 0.002; see Figure S5).

### 3.3 Subjective Ratings

**FIGURE 7:**
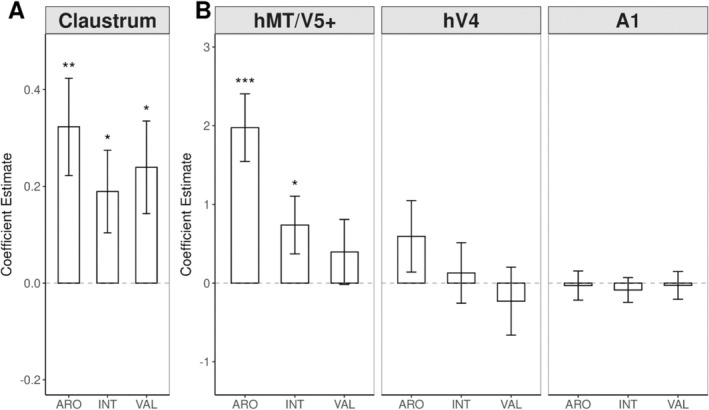
Association with subjective ratings of the videos. (A) Coefficient estimates plotted for the visual claustrum for arousal (ARO), interest (INT) and valence (VAL). (B) Coefficient estimates plotted for the cortical regions hMT/V5+, hV4 and primary auditory cortex (A1) for arousal (ARO), interest (INT) and valence (VAL). Note that each subjective feature (arousal, interest, and absolute valence) was analyzed in a separate linear mixed model.

We performed a linear mixed model analysis with either arousal, interest, or valence as fixed effects and participants as random effects to determine if the visual claustrum responses were associated with any of the subjective measures we collected. Claustrum visual zone activity was positively associated with arousal (Figure 7A; *t*(1148) = 3.21, *p* = 0.001). This effect was also observed in hMT/V5+ (*t*(1148) = 4.58, *p* < 0.001), but not in hV4 (*t*(1100) = 1.31, *p* = 0.19) and not in the primary auditory cortex (*t*(1148) = −0.17, *p* = 0.87; Figure 7A). Claustrum visual zone activity was also associated with interest (*t*(1148) = 2.22, *p* = 0.03), as was hMT/V5+ response (*t*(1148) = 2.01, *p* = 0.05). However, this was not the case for hV4 (*t*(1100) = 0.34, *p* = 0.74) and the primary auditory cortex (*t*(1148) = −0.56, *p* = 0.58; Figure 7B). Finally, we found that the claustrum showed significant association with valence (*t*(1148) = 2.50, *p* = 0.01), whereas none of the other areas showed a significant association with valence (hMT/V5+: *t*(1148) = 0.96, *p* = 0.34; hV4: *t*(1100) = −0.53, *p* = 0.59; the primary auditory cortex: *t*(1148) = −0.17, p = 0.87; Figure 7B). Full statistical results are shown in Table S3.

Session‐specific analyses were conducted to evaluate the stability of the association with subjective ratings of the videos. As in our main analysis, we found that the claustrum showed a significant association with arousal in both sessions (session 1: *t*(572) = 2.18, *p* = 0.03, session 2: *t*(572) = 2.38, *p* = 0.02), while interest and valence did not reach significance. hMT/V5+ also showed a significant association with arousal in both sessions (session 1: *t*(572) = 3.88, *p* < 0.001, session 2: *t*(572) = 3.16, *p* = 0.002; see Figure S6), while interest and valence did not reach significance in either session. Overall, we conclude that our main results for the subjective ratings were consistent across sessions.

The above similarity between the effects of arousal, interest and valence were confirmed by the intercorrelation among the ratings. Subjective ratings of arousal and interest were significantly correlated with each other (*r* = 0.86, *p* < 0.001) as were the subjective ratings of arousal and valence (*r* = 0.85, *p* < 0.001) and interest and valence (*r* = 0.69, *p* < 0.001).

#### 3.3.1 Relationship Between Claustrum and Cortical Responses Across Participants

To determine whether the magnitude of claustrum responses covaried with cortical responses across participants, we examined correlations between mean response amplitudes in the claustrum and visual cortical regions. We found neither a significant association between claustrum and hMT/V5+ responses (*r* = 0.08, *p* = 0.82), nor between claustrum and A1 (*r* = −0.02, *p* = 1.00). A positive trend was observed between claustrum and hV4 responses (*r* = 0.56, *p* = 0.08), although it did not reach significance (Figure S7).

## 4 Discussion

In this study, we determined the stimulus properties that were associated with responses in the previously identified visual claustrum zone. We examined both the physical feature content of the videos as well as the subjective qualities of the videos as perceived by human volunteers. Visual responses of the claustrum were associated with motion as well as with subjective arousal, interest, and valence ratings. This pattern was somewhat similar to responses of hMT/V5+. Crucially, no equivalent association was found for the auditory cortex, ruling out unspecific global arousal effects. Our results underscore the visual drive of the claustrum visual zone, while at the same time pointing to the influences of visual motion and subjective factors in shaping its visual activity.

### 4.1 Physical Features

The visual claustrum zone showed a significant association with motion content in the videos, which was also present in hMT/V5+ and hV4 (though it was not motion‐specific in the latter). This finding is consistent with what is known about claustrum functional organization and claustrocortical connectivity. Specifically, the mammalian claustrum is known to contain distinct sensory zones. These distinct sensory zones receive input from the corresponding sensory cortical regions (Gattass et al. 2014; Jayaraman and Updyke 1979; LeVay and Sherk 1981b, 1981a; Olson and Graybiel 1980), with neurons responding to modality‐specific sensory stimulation (Olson and Graybiel 1980; Remedios et al. 2010, 2014; Sherk and LeVay 1981). This sensory‐specific claustrocortical organization has also been confirmed in humans using diffusion‐weighted MRI (Fernandez‐Miranda et al. 2012; Fernández‐Miranda et al. 2008; Torgerson et al. 2014; Wendt et al. 2024). The visual sensory zone receives input from multiple cortical visual areas, including V4, MT, and V1 (Gattass et al. 2014; LeVay and Sherk 1981b). It is therefore expected that claustrum visual response characteristics will bear some similarity with those of the visual areas it receives projections from. It is also well established that each sensory zone is connected to the corresponding sensory cortical regions, yielding a topographically organized connectivity pattern.

Notably, the claustrum's sensitivity to motion observed in our study aligns well with findings from the cat claustrum, where neurons showed a clear preference for moving stimuli (Sherk and LeVay 1981). In that study, the authors varied the velocity and size of a moving bar stimulus presented in the periphery. Although felines possess a high density of rods (Steinberg et al. 1973), which are highly sensitive to peripheral motion (Gerstner 2011), a characteristic that may have amplified claustrum responses in cats, we nonetheless observed a significant motion effect in the human claustrum using naturalistic video stimulation. This convergence across species and methodologies strengthens the case that motion sensitivity is a fundamental property of the visual claustrum zone. That said, our field‐of‐view inside the 7 T scanner was constrained to the central 14 degrees of eccentricity, which limited the extent of stimulation of the visual periphery. Future studies using wide field of view visual stimulation systems may therefore reveal even stronger motion‐related responses in the human claustrum.

The shared sensitivity to motion across the claustrum, hMT/V5+, and hV4 is consistent with the known claustrocortical connectivity pattern. Visual cortex neurons are primarily driven by low‐level stimulus features such as luminance and contrast (Felleman and Van Essen 1991; Hubel and Wiesel 1959; Tang et al. 2021; Wang, Li, et al. 2015), and motion information could be passed to the visual claustrum zone via these claustrocortical connections (Gardner et al. 2005; Roe et al. 2012; Sani et al. 2013). However, we did not find evidence that the magnitude of claustrum responses covaried with responses in hMT/V5+ or hV4 across participants. This suggests that, although the claustrum and visual cortical areas share sensitivity to motion, claustrum responses are not simply inherited from cortical response strength. While the current sample size may limit sensitivity to detect weaker relationships, the absence of a clear correlation argues against a straightforward feedforward scaling account.

In contrast to the motion effect, the claustrum did not show a significant association with any of the low‐level visual features, including temporal contrast, color intensity, or spatial contrast. This dissociation is noteworthy, as it suggests that the visual claustrum zone is more selectively tuned to motion than to static visual properties. Area hMT/V5+ showed a significant negative association with spatial contrast but not with temporal contrast or color intensity, while hV4 was significantly associated with temporal contrast. Of note, the residual pixel difference metric, which captures luminance changes remaining after accounting for translational motion between frames, was significantly associated with both hMT/V5+ and hV4, yet in opposite directions. The negative association with residual pixel difference in hMT/V5+ may reflect the fact that, after motion is accounted for, the remaining pixel changes represent non‐motion‐related visual variation that is less relevant to this motion‐selective region. In contrast, the positive association in hV4 is consistent with this area's well‐established sensitivity to contrast‐related visual features.

Our fMRI acquisition protocol was optimized for detecting claustrum responses, which prevented us from including the primary visual cortex as well as the LGN in our slice coverage. Future studies would benefit from additionally including these two early visual regions to directly compare their responses with those of the claustrum. Furthermore, an analysis additional analysis modelling the time course of each full time course of the movie feature in a first‐level s using a GLM, such as the one performed by Bartels et al. (2008), may have been more sensitive for capturing the covariation between feature dynamics and claustrum activity; likewise, it yielded weaker and less consistent effects in the claustrum. Such an analysis was not feasible with the current data due to relatively short movie segments interleaved with other conditions as well as the baseline periods. Future studies using longer continuous movie segments should be able to overcome this limitation. This difference likely reflects the distinct properties of the two analytical approaches, as the linear mixed model analysis captures variability in feature responses across videos, whereas the time course‐based GLM models continuous fluctuations in feature content over time. Furthermore, the linear mixed model analysis has superior statistical power, as it additionally considers subject‐level variation.

### 4.2 Subjective Features

We found that the visual claustrum zone showed an association with subjective ratings of emotional arousal, interest, and valence in the natural videos. Moreover, we found that the visual claustrum showed consistent associations with arousal across both sessions. This finding aligns with the claustrum's hypothesized role in selective attention. Emotional arousal is closely tied to attention, as emotionally arousing stimuli demand attentional resources (Fernandes et al. 2011). Moreover, interest, as well as high or low valence, are closely linked to emotional arousal, which may contribute to the demand for attentional resources. The claustrum is hypothesized to have an important role in attention modulation, not least because in addition to sensory regions it receives input from higher‐order brain regions such as the anterior cingulate cortex (ACC) (Goll et al. 2015; Jackson et al. 2020). It is suggested that the claustrum feeds information back to cortical areas so that more attentional resources are given to salient stimuli (Goll et al. 2015; Smith et al. 2019). The claustrum's role as an attention modulator has been extended to include its involvement in, and functional connectivity to, limbic and affective brain regions (Smith et al. 2019). At the cellular level, claustrum neurons express a high density of serotonin 2A receptors (5‐HT2A), which are implicated in modulating attentional focus (Liaw and Augustine 2023), and disruption of these receptors leads to an altered state of attention and perception (Stiefel et al. 2014). Our findings on the association between claustrum activity and the arousing effects of videos further support the claustrum's role in attention in humans.

We additionally found that the visual claustrum zone showed an association with subjective ratings of interest and valence to the natural videos. Arousal, interest and valence were themselves correlated, suggesting a common underlying process related to motivational salience or attentional engagement. Consistent with the perspective that emotional arousal modulates attention, the interest of an individual in a stimulus is associated with patterns of activity across large‐scale neural networks that support salience detection and attentional control (Di Domenico and Ryan 2017). More specifically, increased curiosity, which is a concept closely related to interest, has been shown to increase activity in the ACC (Jepma et al. 2012), an area with abundant connections with the claustrum. The more interesting and emotionally arousing naturalistic stimuli are likely to cause claustrum‐targeted feedback from frontal areas for example, ACC that are driven by emotional arousal, attentional demands and interest.

Interestingly, hMT/V5+ also showed a preference for arousal and interest. This aligns with a broader understanding that emotional arousal significantly modulates activity throughout the visual cortex (Lang et al. 1998). The impact of emotion appears particularly pronounced in higher‐order visual areas, which are involved in more complex visual processing. More recently, increased hMT/V5+ activity has been found when comparing the brain activity for aversive versus neutral images (Fourcade et al. 2022). With aversive stimuli being more arousal‐inducing than neutral stimuli, the study showed that all higher‐order visual areas were activated during more arousal‐inducing stimuli and this effect held when matching the physical features content of the images (Taylor et al. 2000). The neural mechanisms underlying this emotional modulation of visual processing are thought to involve feedback projections from limbic structures, such as the amygdala, to visual cortical areas (Price 2003; Tamietto et al. 2012). The amygdala is known to respond strongly to emotionally arousing stimuli and is well‐positioned to influence sensory processing based on affective relevance (Hadj‐Bouziane et al. 2012; Pessoa et al. 2006). It could therefore influence visual areas and the visual claustrum zone in parallel.

Crucially, we did not find an association between subjective ratings and activity in the primary auditory cortex. We can therefore rule out the possibility that the effects of arousal and interest observed in visual areas and the claustrum were driven by unspecific physiological arousal that can influence global fMRI activity. There is a clear distinction between physiological arousal and emotional arousal, whereby only the latter can modulate attention (Zsidó 2024).

## 5 Conclusion

We found that the responses in the visual claustrum zone in humans are associated with visual motion on the one hand and with subjective arousal, interest, and valence of the stimuli on the other hand. Our results provide support for the idea that the claustrum is involved in sensory processing which goes beyond simple sensory feature extraction. Additionally, they support the visual claustrum's involvement in saliency processing and attention. Further research is needed to confirm these findings by experimentally manipulating saliency and attention.

## Author Contributions

A.C. contributed to conceptualization, data analysis, original draft writing, reviewing and editing, and methodology. P.S., A.W. and H.M. and D.L. contributed to data collection, methodology and review and editing. C.W. and A.B. contributed to review and editing and methodology. A.I. contributed to supervision, review and editing. N.Z. contributed to conceptualization, funding resources, project administration, resources, methodology, supervision, and review and editing.

## Funding

This research was funded in whole or in part by the Austrian Science Fund (FWF) [10.55776/P35583; 10.55776/PAT8722623]. For open access purposes, the author has applied a CC BY public copyright license to any author‐accepted manuscript version arising from this submission. This research was supported by a BioTechMed‐Graz Young Researcher Group Grant to N.Z., and by funding within the framework of the Dimitrov Fellowship of the OeAW to A.C.

## Conflicts of Interest

The authors declare no conflicts of interest.

## Supporting information


**Figure S1:** Quality of coregistration. Each subject with either left or right claustrum scanned. Images show the structural scan with EPI overlaid and with grey and white matter boundaries highlighted. Yellow ROI indicates the visual claustrum zone.
**Figure S2:** Temporal signal‐to‐noise ratio in the claustrum, putamen, insula, and the visual regions included in the analysis (hMT/V5+ and hV4) and the control region primary auditory cortex (A1). (A) TSNR maps of a representative subject with left claustrum scanned, with ROIs shown as outlines. (B) TSNR maps of representative subject with right claustrum scanned. (C) Average tSNR bar plot for each region. Individual points represent single subjects. Error bars represent SEM.
**Figure S3:** Claustrum FIR response compared with the predicted response based on the canonical HRF. Group‐level finite impulse response (FIR) time course of the visual claustrum during visual blocks plotted against the predicted response derived by the convolution of the stimulus boxcar function with the canonical SPM HRF. For better comparability, both curves were normalized to a peak of 1.
**Table S1:** Linear mixed model results for each region using temporal contrast (tCON), color (COL) and spatial contrast (sCON) features as fixed effects and subjects modelled as a random effect.
**Figure S4:** Responses to low‐level features for session 1 (grey) and session 2 (white). Linear mixed model analysis including temporal contrast (tCON), color (COL) and spatial contrast (sCON). Coefficient estimates plotted for (A) the visual claustrum and (B) cortical regions hMT/V5+, hV4 and primary auditory cortex (A1).
**Table S2:** Linear mixed model results for each region using motion (MOT) and residual (RES) features as fixed effects and subjects modelled as a random effect.
**Figure S5:** Responses to motion and residuals for session 1 (grey) and session 2 (white). Linear mixed model analysis including motion (MOT) and residual (RES) (pixel differences not explained by motion). Coefficient estimates plotted for (A) the visual claustrum and (B) cortical regions hMT/V5+, hV4 and primary auditory cortex (A1).
**Table S3:** Linear mixed methods results for each region using arousal (ARO), interest (INT) and valence (VAL) subjective ratings as fixed effects and subjects modelled as a random effect.
**Figure S6:** Association with subjective ratings of the videos using the fMRI data from session 1 (grey) and session 2 (white). (A) Coefficient estimates plotted for the visual claustrum for arousal (ARO), interest (INT) and valence (VAL). (B) Coefficient estimates plotted for the cortical regions hMT/V5+, hV4 and primary auditory cortex (A1) for arousal (ARO), interest (INT) and valence (VAL). Note, each subjective feature (arousal, interest, and absolute valence) was analyzed in a separate linear mixed model.
**Figure S7:** Relationship between claustrum responses and cortical responses across participants. Scatter plots show an association between claustrum responses and responses in (A) hMT/V5+, (B) hV4, and (C) A1. Each point represents one participant; solid lines indicate least‐squares regression fits with 95% confidence intervals. Correlation coefficients and associated *p* values are shown in each panel. No significant association was observed between claustrum responses and any of the comparison regions.

## Data Availability

The data that support the findings of this study are openly available in Open Science Framework at https://doi.org/10.17605/OSF.IO/WPA4V.
