# Supplementary material for: The Human Visual Claustrum Responses to Physical Stimulus Properties and Subjective Content During Movie Viewing
Source: Hum Brain Mapp. 2026 Jun 25;47(9):e70583. doi: 10.1002/hbm.70583 (PMC13303107; doi:10.1002/hbm.70583)
Supplement: Supplementary file 1 — Figure S1: Quality of coregistration. Each subject with either left or right claustrum scanned. Images show the structural scan with EPI overlaid and with grey and white matter boundaries highlighted. Yellow ROI indicates the visual claustrum zone. Figure S2: Temporal signal‐to‐noise ratio in the claustrum, putamen, insula, and the visual regions included in the analysis (hMT/V5+ and hV4) and the control region primary auditory cortex (A1). (A) TSNR maps of a representative subject with left claustrum scanned, with ROIs shown as outlines. (B) TSNR maps of representative subject with right claustrum scanned. (C) Average tSNR bar plot for each region. Individual points represent single subjects. Error bars represent SEM. Figure S3: Claustrum FIR response compared with the predicted response based on the canonical HRF. Group‐level finite impulse response (FIR) time course of the visual claustrum during visual blocks plotted against the predicted response derived by the convolution of the stimulus boxcar function with the canonical SPM HRF. For better comparability, both curves were normalized to a peak of 1. Table S1: Linear mixed model results for each region using temporal contrast (tCON), color (COL) and spatial contrast (sCON) features as fixed effects and subjects modelled as a random effect. Figure S4: Responses to low‐level features for session 1 (grey) and session 2 (white). Linear mixed model analysis including temporal contrast (tCON), color (COL) and spatial contrast (sCON). Coefficient estimates plotted for (A) the visual claustrum and (B) cortical regions hMT/V5+, hV4 and primary auditory cortex (A1). Table S2: Linear mixed model results for each region using motion (MOT) and residual (RES) features as fixed effects and subjects modelled as a random effect. Figure S5: Responses to motion and residuals for session 1 (grey) and session 2 (white). Linear mixed model analysis including motion (MOT) and residual (RES) (pixel differences not explained by motion) [file HBM-47-e70583-s001.docx]

The human visual claustrum responses to physical stimulus properties and subjective content during movie viewing

Supplementary Material

Adam Coates¹^,2^*, Paul Sedlmayr¹, Anna Wastian¹, Hannes Mayrhofer¹, David Linhardt^3^, Christian Windischberger^3^, Andreas Bartels^4^, Anja Ischebeck¹, Natalia Zaretskaya¹^,2^

¹ Department of Psychology, University of Graz, Graz, Austria

^2^ BioTechMed-Graz, Graz, Austria

^3^ High Field MR Center, Center for Medical Physics and Biomedical Engineering, Medical University of Vienna, Vienna, Austria

^4^ Werner Reichardt Centre for Integrative Neuroscience, University of Tübingen, Tübingen, Germany

*Corresponding author: [adam.coates@uni-graz.at](mailto:adam.coates@uni-graz.at)


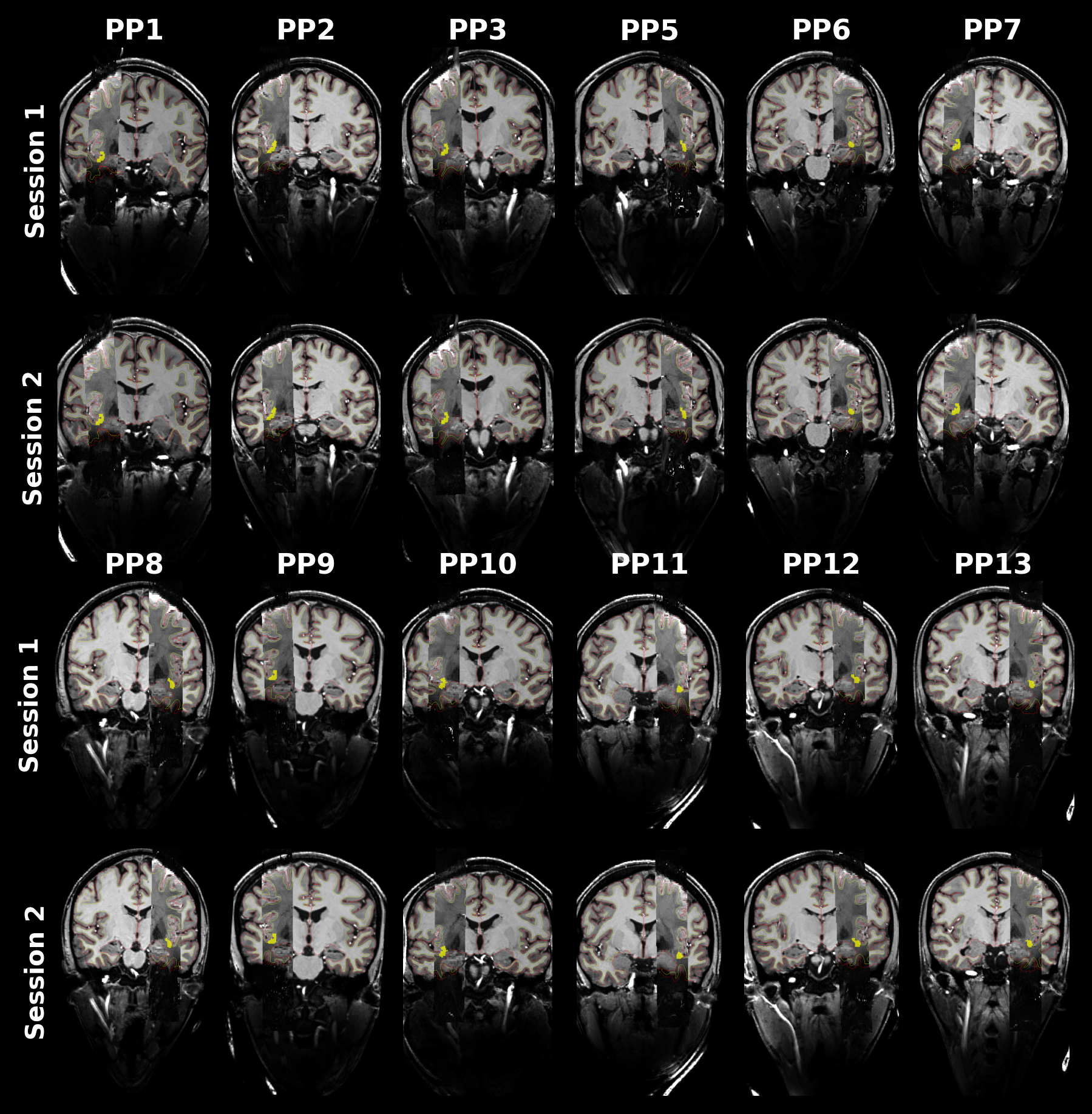


Figure S1. Quality of coregistration. Each subject with either left or right claustrum scanned. Images show the structural scan with EPI overlaid and with grey and white matter boundaries highlighted. Yellow ROI indicates the visual claustrum zone.


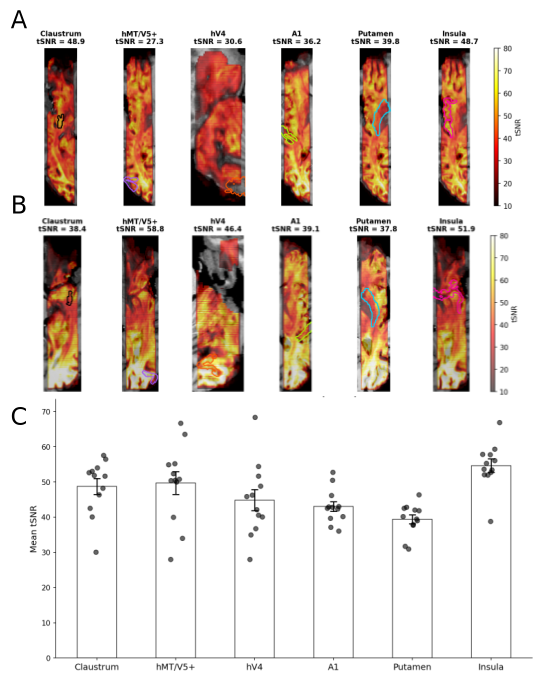


*Figure S2. Temporal signal-to-noise ratio in the claustrum, putamen, insula, and the visual regions included in the analysis (hMT/V5+ and hV4) and the control region primary auditory cortex (A1). A) TSNR maps of a representative subject with left claustrum scanned, with ROIs shown as outlines. B) TSNR maps of representative subject with right claustrum scanned. C) Average tSNR bar plot for each region. Individual points represent single subjects. Error bars represent SEM.*


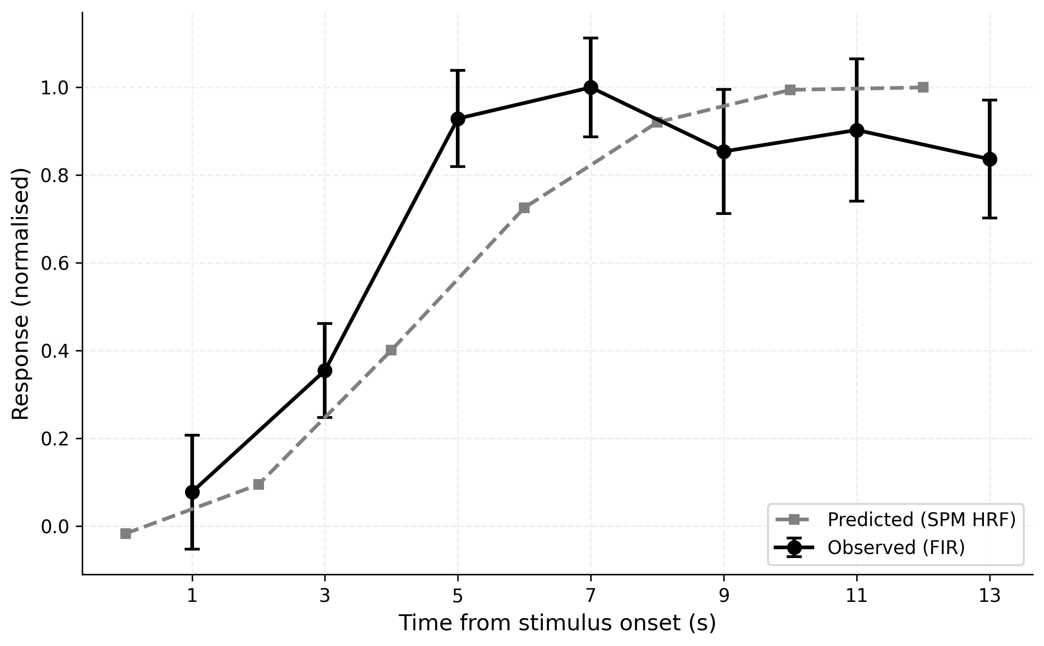


Figure S3. Claustrum FIR response compared with the predicted response based on the canonical HRF. Group-level finite impulse response (FIR) time course of the visual claustrum during visual blocks plotted against the predicted response derived by the convolution of the stimulus boxcar function with the canonical SPM HRF. For better comparability, both curves were normalized to a peak of 1.

Table S1: Linear mixed model results for each region using temporal contrast (tCON), color (COL) and spatial contrast (sCON) features as fixed effects and subjects modelled as a random effect

s

| **Brain Region** | **Predictor** | **Beta** | **95% CI** | **Std.** | **df** | **t** | **p** |
| --- | --- | --- | --- | --- | --- | --- | --- |
| **Claustrum** | tCON | 0.03 | [–0.04, 0.10] | 0.03 | 1146 | 0.90 | 0.368 |
|  | COL | –0.04 | [–0.11, 0.03] | –0.03 | 1146 | –1.09 | 0.275 |
|  | sCON | –0.04 | [–0.11, 0.04] | –0.03 | 1146 | –1.03 | 0.304 |
| **hMT/V5+** | tCON | 0.03 | [–0.28, 0.33] | 0.004 | 1146 | 0.17 | 0.865 |
|  | COL | –0.17 | [–0.48, 0.15] | –0.03 | 1146 | –1.04 | 0.297 |
|  | sCON | –0.43 | [–0.75, –0.11] | –0.07 | 1146 | –2.64 | **0.008** |
| **hV4** | tCON | 0.93 | [0.61, 1.24] | 0.16 | 1098 | 5.84 | **< .001** |
|  | COL | –0.31 | [–0.63, 0.02] | –0.05 | 1098 | –1.84 | 0.066 |
|  | sCON | –0.11 | [–0.44, 0.21] | –0.02 | 1098 | –0.68 | 0.497 |
| **A1** | tCON | –0.05 | [–0.18, 0.08] | –0.02 | 1146 | –0.74 | 0.457 |
|  | COL | –0.005 | [–0.14, 0.13] | –0.002 | 1146 | –0.07 | 0.944 |
|  | sCON | –0.03 | [–0.16, 0.11] | –0.01 | 1146 | –0.39 | 0.695 |


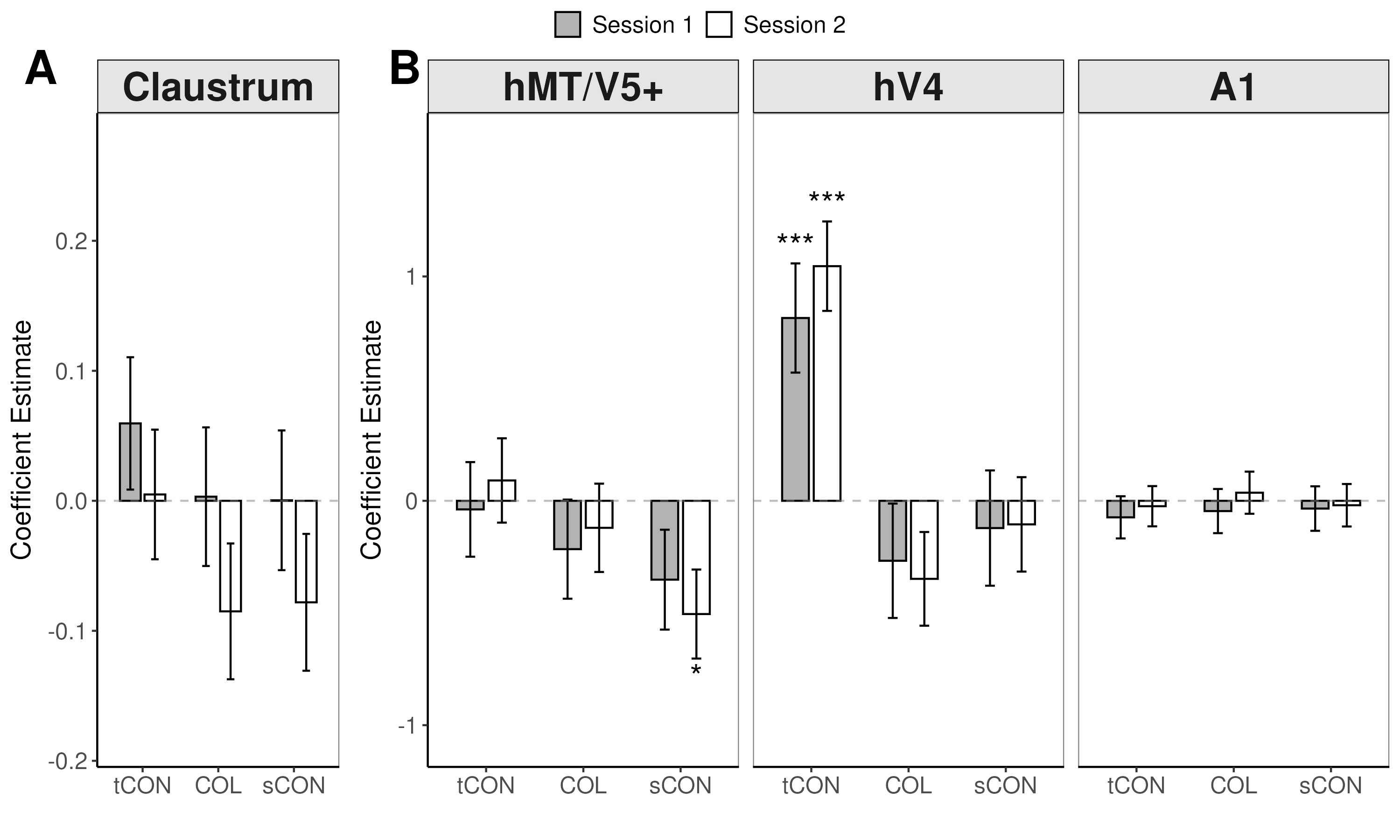


Figure S4. Responses to low-level features for session 1 (grey) and session 2 (white). Linear mixed model analysis including temporal contrast (tCON), color (COL) and spatial contrast (sCON). Coefficient estimates plotted for A) the visual claustrum and B) cortical regions hMT/V5+, hV4 and primary auditory cortex (A1).

Table S2: Linear mixed model results for each region using motion (MOT) and residual (RES) features as fixed effects and subjects modelled as a random effect.

| **Brain Region** | **Predictor** | **Beta** | **95% CI** | **Std.** | **df** | **t** | **p** |
| --- | --- | --- | --- | --- | --- | --- | --- |
| **Claustrum** | MOT | 0.10 | [0.01, 0.18] | 0.08 | 1147 | 2.27 | **0.024** |
|  | RES | –0.04 | [–0.12, 0.04] | –0.03 | 1147 | –0.97 | 0.334 |
| **hMT/V5+** | MOT | 0.95 | [0.59, 1.30] | 0.16 | 1147 | 5.26 | **< .001** |
|  | RES | –0.71 | [–1.06, –0.35] | –0.12 | 1147 | –3.94 | **< .001** |
| **hV4** | MOT | 0.63 | [0.27, 1.00] | 0.11 | 1099 | 3.39 | **< .001** |
|  | RES | 0.47 | [0.11, 0.84] | 0.08 | 1099 | 2.54 | **0.011** |
| **A1** | MOT | –0.15 | [–0.30, 0.005] | –0.07 | 1147 | –1.89 | 0.059 |
|  | RES | 0.05 | [–0.10, 0.20] | 0.02 | 1147 | 0.67 | 0.503 |


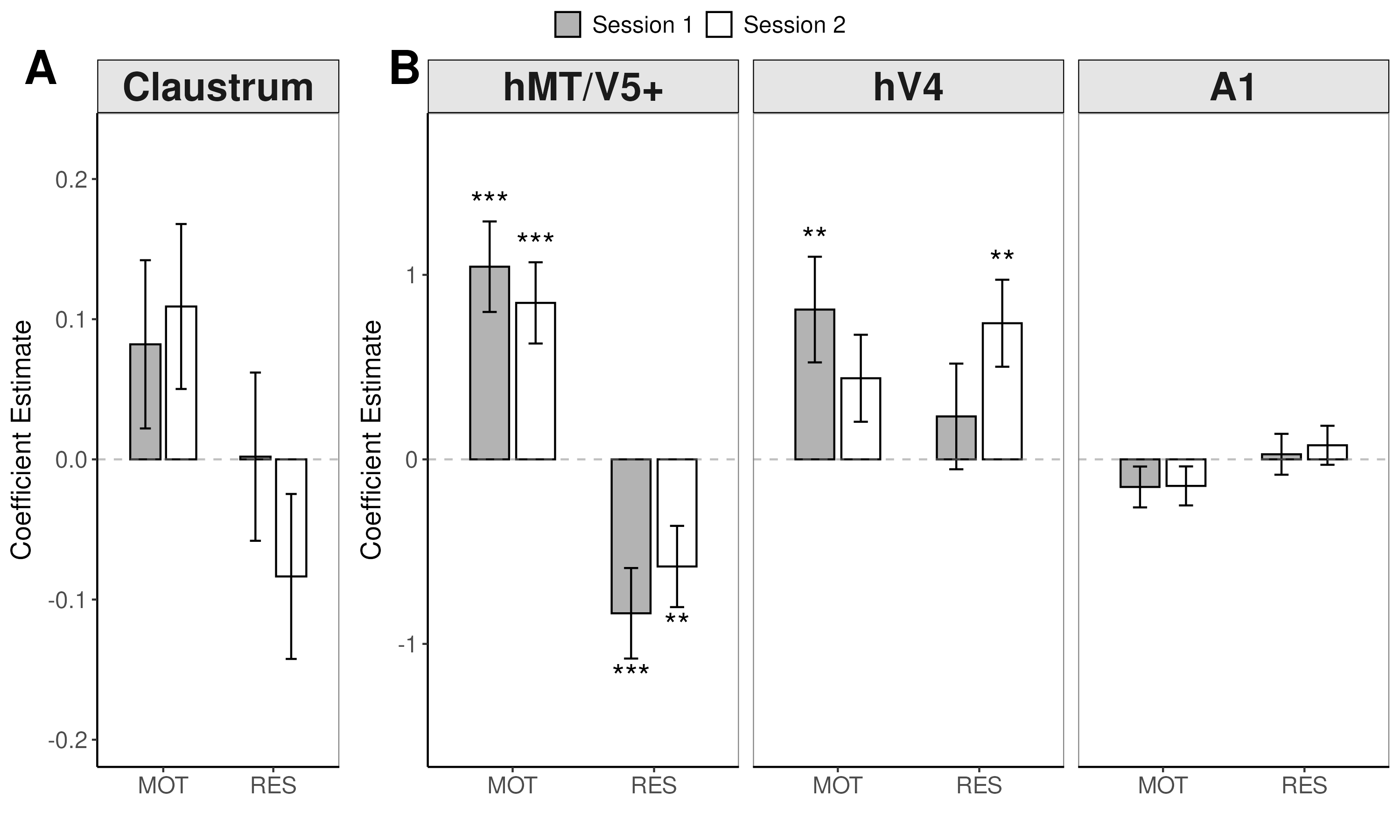


Figure S5. Responses to motion and residuals for session 1 (grey) and session 2 (white). Linear mixed model analysis including motion (MOT) and residual (RES) (pixel differences not explained by motion). Coefficient estimates plotted for A) the visual claustrum and B) cortical regions hMT/V5+, hV4 and primary auditory cortex (A1).

Table S3: Linear mixed methods results for each region using arousal (ARO), interest (INT) and valence (VAL) subjective ratings as fixed effects and subjects modelled as a random effect.

| **Predictor** | **Brain Region** | **Beta** | **95% CI** | **Std.** | **df** | **t** | **p** |
| --- | --- | --- | --- | --- | --- | --- | --- |
| **Arousal** | Claustrum | 0.32 | [0.13, 0.52] | 0.09 | 1148 | 3.21 | **0.001** |
|  | hMT/V5+ | 1.98 | [1.13, 2.82] | 0.12 | 1148 | 4.58 | **< .001** |
|  | hV4 | 0.59 | [–0.30, 1.48] | 0.04 | 1100 | 1.31 | 0.192 |
|  | A1 | –0.03 | [–0.39, 0.33] | –0.005 | 1148 | –0.17 | 0.868 |
| **Interest** | Claustrum | 0.19 | [0.02, 0.36] | 0.06 | 1148 | 2.22 | **0.027** |
|  | hMT/V5+ | 0.74 | [0.02, 1.46] | 0.05 | 1148 | 2.01 | **0.045** |
|  | hV4 | 0.13 | [–0.63, 0.88] | 0.009 | 1100 | 0.34 | 0.737 |
|  | A1 | –0.09 | [–0.40, 0.22] | –0.02 | 1148 | –0.56 | 0.577 |
| **Valence** | Claustrum | 0.24 | [0.05, 0.43] | 0.07 | 1148 | 2.50 | **0.012** |
|  | hMT/V5+ | 0.40 | [–0.41, 1.21] | 0.03 | 1148 | 0.96 | 0.338 |
|  | hV4 | –0.23 | [–1.08, 0.62] | –0.01 | 1100 | –0.53 | 0.594 |
|  | A1 | –0.03 | [–0.37, 0.32] | –0.005 | 1148 | –0.17 | 0.869 |


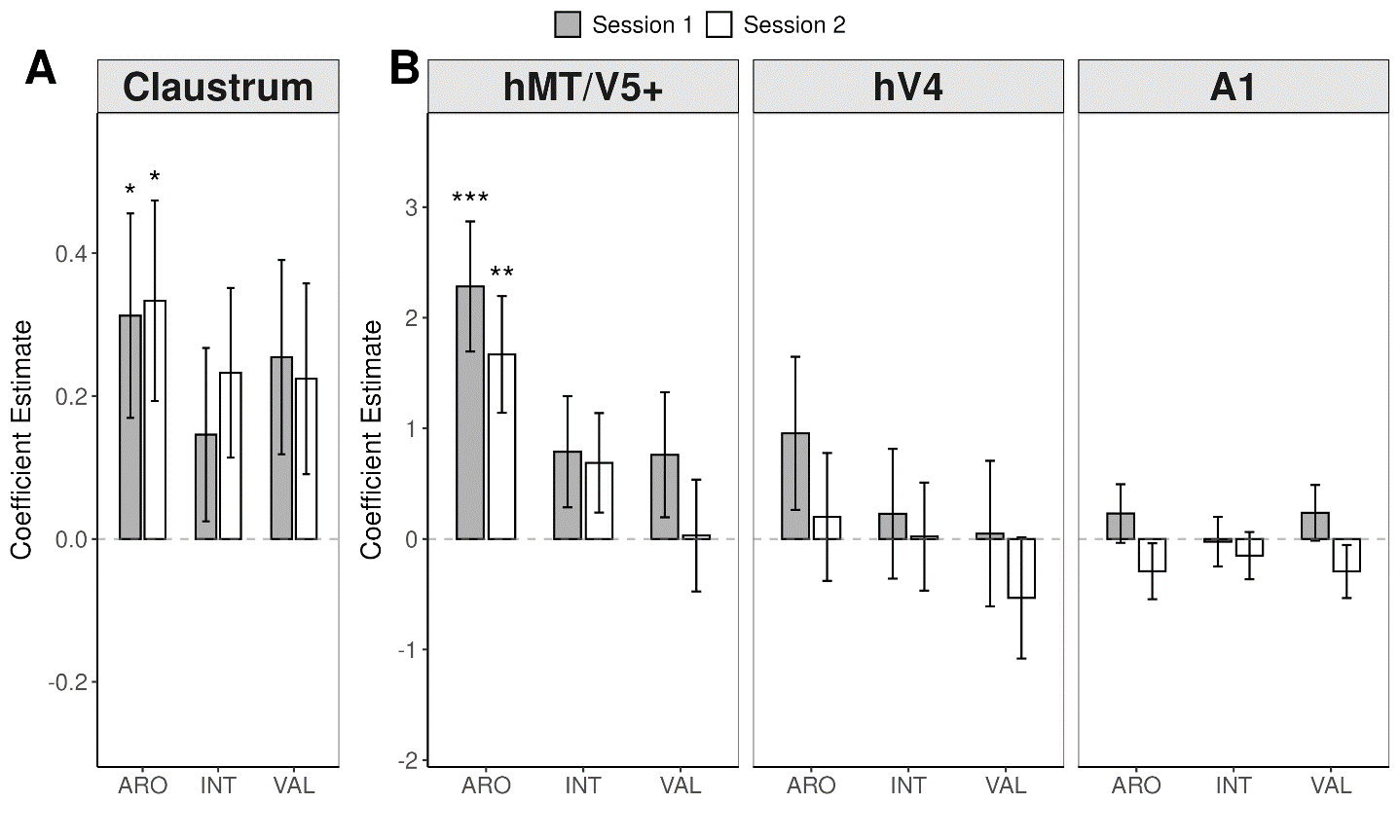


Figure S6. Association with subjective ratings of the videos using the fMRI data from session 1 (grey) and session 2 (white). A) Coefficient estimates plotted for the visual claustrum for arousal (ARO), interest (INT) and valence (VAL). B) Coefficient estimates plotted for the cortical regions hMT/V5+, hV4 and primary auditory cortex (A1) for arousal (ARO), interest (INT) and valence (VAL). Note, each subjective feature (arousal, interest, and absolute valence) was analyzed in a separate linear mixed model.


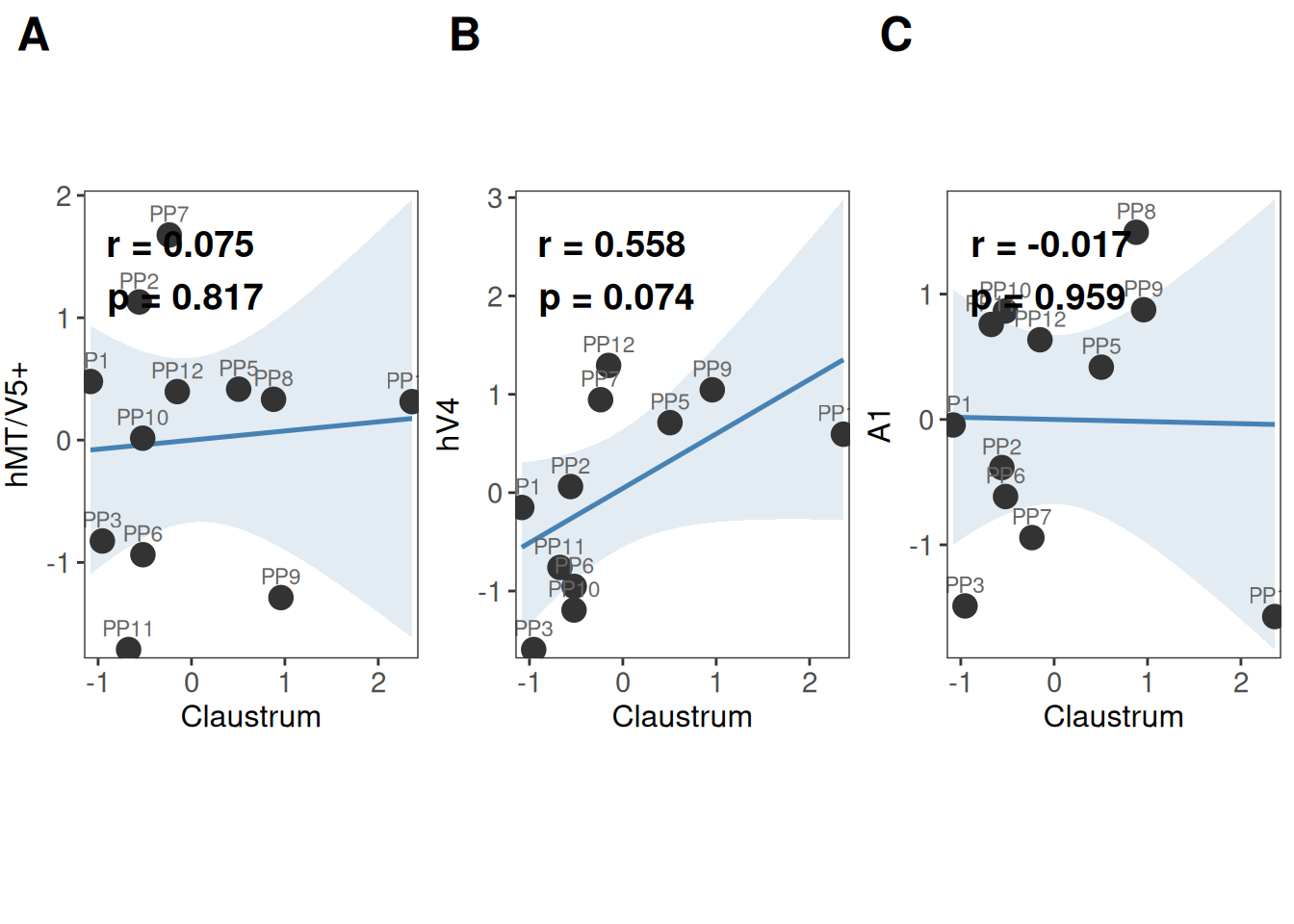


Figure S7. Relationship between claustrum responses and cortical responses across participants. Scatter plots show an association between claustrum responses and responses in (A) hMT/V5+, (B) hV4, and (C) A1. Each point represents one participant; solid lines indicate least-squares regression fits with 95% confidence intervals. Correlation coefficients and associated p values are shown in each panel. No significant association was observed between claustrum responses and any of the comparison regions.
